# PDK1 promotes epithelial ovarian cancer progression by upregulating BGN: PDK1/BGN axis facilitates EOC progression

**DOI:** 10.3724/abbs.2024186

**Published:** 2024-11-21

**Authors:** Lei Zhang, Lina Yan, Xin Fu, Ziqi Tao, Shuna Liu, Rong Li, Ting Wang, Yepeng Mao, Wenwen Shang, Mi Gong, Xuemei Jia, Fang Wang

**Affiliations:** 1 Department of Laboratory Medicine the First Affiliated Hospital with Nanjing Medical University Nanjing 210029 China; 2 Branch of National Clinical Research Center for Laboratory Medicine Nanjing 210029 China; 3 Department of Gynecology the Affiliated Huaian No. 1 People’s Hospital of Nanjing Medical University Huaian 223300 China; 4 Department of Gynecology Women’s Hospital of Nanjing Medical University Nanjing 210004 China; 5 Clinical Laboratory Baoshan People’s Hospital Baoshan 678000 China

**Keywords:** epithelial ovarian cancer, PDK1, BGN, EMT, NF-κB

## Abstract

Pyruvate dehydrogenase kinase 1 (PDK1) is a new therapeutic target that is dysregulated in multiple tumors. This study aims to explore the potential role and regulatory mechanism of PDK1 in epithelial ovarian cancer (EOC). We detect PDK1 expression in EOC tissues and cells using qRT-PCR and western blot analysis, and the effects of PDK1 on EOC cell malignant behaviors are explored. RNA sequencing analyses are performed to explore the differentially expressed genes in *PDK1*-silenced EOC cells. Furthermore, tumor-bearing mouse models are established to assess the impacts of PDK1 and BGN on EOC tumor growth and metastasis *in vivo*. The results show that PDK1 is upregulated in EOC tissues and cell lines. Biglycan (BGN) is downregulated in *PDK1*-silenced EOC cells, and its expression is positively correlated with PDK1 levels in EOC tissues. PDK1 depletion inhibits EOC cell proliferation, migration and invasion. Mechanistically, PDK1 and BGN are colocalized in the cytoplasm of EOC cells and interact with each other. PDK1 positively regulates BGN expression by enhancing *BGN* mRNA stability. BGN overexpression partially reverses the anti-tumor effects of PDK1 depletion on EOC cell malignant behaviors. PDK1 has also been revealed to upregulate BGN to activate the NF-κB oncogenic pathway in EOC cells. Additionally, PDK1 accelerates tumor growth and metastasis by modulating BGN expression. In conclusion, *PDK1* functions as an oncogene, facilitating EOC progression by upregulating BGN and activating the NF-κB pathway. These findings may provide valuable biomarkers for the diagnosis and treatment of EOC.

## Introduction

Epithelial ovarian cancer (EOC) is one of the most common malignant tumors of the female reproductive system and severely endangers the life and health of women worldwide [1]. Because of the lack of specific symptoms and early detection methods, EOC patients are often diagnosed with advanced-stage disease [2], but the 5-year survival rate at the late stage is lower than 30% [3]. CA125 and HE4 are valuable diagnostic biomarkers for ovarian cancer, especially for evaluating chemotherapeutic efficacy and clinical outcomes. However, these biomarkers remain insufficiently reliable for screening early-stage ovarian cancer for diagnosis [ 4, 5] . Moreover, the treatment of ovarian cancer is limited by chemoresistance and recurrence, and targeted therapy is suggested to have clinical benefits [ 6, 7] . Hence, understanding the molecular mechanism of EOC and exploring promising diagnostic biomarkers and therapeutic targets for EOC are essential.

The identification of novel therapeutic targets easily modulated by small molecules contributes to targeted therapy in cancer. PDK1 is abnormally expressed in various cancers and is involved in cancer progression. For example, *PDK1* silencing suppresses breast cancer cell migration and invasion [8]. PDK1 facilitates hypopharyngeal carcinoma metastasis by inducing epithelial-mesenchymal transition (EMT) via the Notch1 pathway [9]. PDK1 is highly expressed in hepatocellular carcinoma (HCC) and enhances the resistance of HCC cells to radiotherapy by activating PI3K signaling; promoting HCC cell migration, invasion, and EMT; and enhancing cancer cell stemness [10]. PDK1 is upregulated in non-small cell lung cancer (NSCLC) cells, and *PDK1* silencing inhibits the survival of NSCLC cells via the Hippo-YAP/IRS2 pathway [11]. Additionally, PDK1 is negatively correlated with CD8 ^+^ T-cell infiltration in ovarian cancer, and *PDK1* knockdown is linked to improved CD8 ^+^ T-cell function and survival in tumor-bearing mice [12]. On the basis of our previous report, the *PDK1* gene has significant clinical and prognostic value in the management of EOC. EOC patients with lower PDK1 expression have a higher overall survival rate [13]. This evidence implies that PDK1 may play a critical role in EOC, and the effects and underlying mechanisms of PDK1 targeting are expected to be further explored.

Biglycan (BGN), a constituent of the small leucine-rich proteoglycan family, plays a vital role in modulating cellular adhesion, migration, cell proliferation, autophagy, inflammation, survival, cell morphology, and motility [ 14‒ 18] . Previous studies have demonstrated the high expression of BGN in various tumor tissues [ 19‒ 21] . Moreover, *BGN* has been reported to be an oncogene in a variety of cancers, including pancreatic, esophageal, gastric and endometrial cancer [ 22, 23] . In addition, BGN reportedly enhances the migration and invasion of endometrial cancer cells and is proposed as a promising EMT biomarker for colorectal cancer [ 24, 25] . BGN is also significantly correlated with EMT gene signatures in bladder cancer, and EMT is closely associated with BGN-driven oncogenesis [26]. Nevertheless, the specific contribution of BGN to EOC remains unclear.

Herein, we focused on the potential role of PDK1 and BGN in EOC. The molecular mechanism of PDK1 in EOC was explored, and BGN was identified as the downstream target of PDK1 and served as a promising serum biomarker for EOC diagnosis. PDK1 promoted the progression of EOC by upregulating BGN, which might offer potential therapeutic targets for EOC.

## Materials and Methods

### Cells and culture

Human EOC cell lines (ES-2, A2780, Caov3 and Skov3), as well as the normal ovary epithelial cell line Hosepic, were purchased from the Cell Bank of the Chinese Academy of Sciences (Shanghai, China). The cells were maintained in McCoy’s 5A (Invitrogen, Carlsbad, USA) or DMEM (Gibco, Carlsbad, USA) supplemented with 10% fetal bovine serum (FBS; Gibco), 100 μg/mL penicillin and 100 U/mL streptomycin at 37°C with 5% CO _2_.

### Construction of stable cell lines

*PDK1*-silenced lentiviruses (shPDK1), *BGN*-overexpressing lentiviruses (oeBGN) and empty vector virus (control) were obtained from Gene Pharma (Shanghai, China). Caov3 and Skov3 cells (5 × 10 ^5^/mL) were cultured overnight, and a proper amount of virus was added on the basis of the MOI of the cell line. The sequences used to contrast shPDK1 were as follows: shPDK1-1, 5′-GCTGAGGATGCTAAAGCTATT-3′; shPDK1-2, 5′-GCATAAATCCAAACTGCAATG-3′; shPDK1-3, 5′-GCGTCTGTGTGATTTGTATTA-3′; and shPDK1-4, 5′-GCAGAGAACCCAAAGACATGA-3′. The sequence used as a negative control was as follows: shNC, 5′-GAAGTGGCTAGACCTGACGCTAGG-3′. Following transduction, the cells were selected in medium containing 1.5 μg/mL puromycin for 14 days to establish stable cell lines.

### Quantitative real-time PCR (qRT-PCR)

The mRNA levels were analyzed using qRT-PCR as previously reported [13]. The oligonucleotide primers used were synthesized by Genscript (Nanjing, China) and the sequences are as follows: *PDK1* (forward: 5′-CTGTGATACGGATCAGAAACCG-3′; reverse: 5′-TCCACCAAACAATAAAGAGTGCT-3′); *BGN* (forward: 5′-GGGTCTCCAGCACCTCTACGC-3′; reverse: 5′-TGAACACTCCCTTGGGCACCT-3′); and *β-actin* (forward: 5′-GAGCTACGAGCTGCCTGACG-3′; reverse: 5′-GTAGTTTCGTGGATGCCACAG-3′).

### Western blot analysis

Western blot analysis was performed as previously reported [27]. The primary antibodies used included anti-PDK1 (ab110025; Abcam, Cambridge, UK), anti-BGN (ab226991; Abcam), anti-p65 (#8242; Cell Signaling Technology, Danvers, USA), anti-p-p65 (#3033; Cell Signaling Technology) and anti-GAPDH (AF0006; Beyotime, Shanghai, China). Anti-E-cadherin, anti-N-cadherin, anti-vimentin and anti-ZEB1 antibodies were obtained from Cell Signaling Technology. The secondary antibodies were obtained from Beyotime (Shanghai, China).

### Cell proliferation assay

EOC cell proliferation was assessed by Cell Counting kit 8 (CCK 8) assay. Cells were seeded in 96-well plates at a density of 5 × 10 ^3^/ well. CCK-8 assays were performed according to the manufacturer’s instructions. Briefly, 10 μL of CCK-8 solution (Beyotime) was added to each well of different plates at 24, 48, 72, and 96 h, and cells were incubated with this solution for 1.5 h. The optical density (OD) was measured at 450 nm using a microplate reader (Biotek, Winooski, USA). For the colony formation assay, the cells were plated in 6-well plates and cultured for 14 days. Following fixation, the cells were stained with 0.1% crystal violet and the number of colonies was counted.

### Wound healing and transwell assays

For the wound-healing assay, the cells were grown in a 6-well plate to 80% confluency. A pipette tip was used to create an artificial wound on the cell monolayer. Pictures were taken under an inverted microscope with the same field of view at 0 h, 24 h and 48 h, and the scratch width was measured. In addition, cell migration and invasion were also determined with Transwell inserts (8 μm; Corning, New York, USA). EOC cells were resuspended, counted and added to the upper chambers with serum-free medium, whereas the lower chamber was filled with medium containing 20% FBS. After incubation for 24 h, the migrated cells on the lower surfaces of the filters were fixed and stained. Similarly, cells were assessed for invasion via the same procedures with the help of Matrigel (BD Biosciences, Franklin Lakes, USA) precoated on the top chambers.

### RNA sequencing (RNA-Seq) analysis

Total RNA from the *PDK1*-silenced group and control group was extracted for RNA sequencing analysis, with three repeated biological samples in each group. The subsequent RNA-Seq analysis was performed by BGI (Shenzhen, China). Genes were identified as significantly differentially expressed when the log2(fold change) was ≥ 1 and the adjusted *P* value was ≤ 0.05. Gene Ontology (GO) and Kyoto Encyclopedia of Genes and Genomes (KEGG) analysis of differentially expressed genes between the *PDK1*-silenced group and control group were obtained from the above RNA-Seq analysis. Ownership of the sequencing data was retained, and data were not submitted to the GEO public database.

### Patients and tissue samples

Sixty EOC patients and 24 control individuals were recruited from the First Affiliated Hospital of Nanjing Medical University and the Affiliated Huai’an No.1 People’s Hospital of Nanjing Medical University. The study was approved by the Hospital Ethics Committee, and all participants signed informed consent forms.

### Immunohistochemistry (IHC)

Paraffin-embedded tissue sections from EOC specimens were dewaxed and rehydrated, and endogenous peroxidase activity was blocked with 3% hydrogen peroxide, followed by antigen recovery. After washing, the sections were incubated with antibodies against PDK1 (1:100; ab110025, Abcam), BGN (1:100; ab226991; Abcam) and Ki-67 (1:150; ab16667; Abcam) at 4°C overnight. After washing, the sections were incubated with the HRP-conjugated second antibody at 37°C for 30 min, followed by visualization with 3,3′-diaminobenzidine tetrahydrochloride (DAB) and counterstaining with hematoxylin. The sections were scored according to their staining intensity and percentage of positive areas [13]. The results were assessed by 2 pathologists who were blinded to the pathological information of the patients.

### Coimmunoprecipitation (Co-IP)

Lysates from Skov3 or Caov3 cells were collected and incubated with the targeted antibodies (or goat-anti-rabbit IgG as a control) and A/G agarose beads (Thermo Fisher Scientific, Waltham, USA) at 4°C for 6 h. After washing, the samples were subjected to western blot analysis.

### RNA fluorescence *in situ* hybridization (FISH) assay

Skov3 or Caov3 cells were treated with 4% formaldehyde for 10 min. After washing with PBS, the cells were dehydrated with ethanol. Next, the cells were incubated with prehybridization solution for 30 min at 37 °C, followed by incubation with specific FISH probes for PDK1 and BGN (Ribobio, Guangzhou, China) in hybridization buffer overnight. After the samples were washed with PBS, DAPI (Beyotime) was used to stain the nuclei. Then, images were captured with a confocal microscope (Leica, Wetzlar, Germany).

### mRNA stability assay

To evaluate the mRNA stability of BGN in EOC cells, transfected Skov3 or Caov3 cells were treated with 2 μg/mL actinomycin D (ActD) (Sigma, St Louis, USA) for 0, 2, 4, 6, or 8 h. TRIzol (Invitrogen) was subsequently used to extract total RNA from the cells, and qRT-PCR was used to detect *BGN* expression.

### *In vivo* experiments

All animal experiments were approved and performed following the guidelines of the Ethics Committee of Nanjing Medical University, and all operations conformed to Animal Ethics Guidelines. A subcutaneous tumor model was established in female BALB/c nude mice (GemPharmatech LLC., Nanjing, China) at 6 weeks of age. Skov3 cells (5 × 10 ^6^ cells) transfected with lentivirus for shPDK1 or control infection or coinfected with shPDK1 + oeBGN were injected into the left axillae. The tumor volume was measured every 3 days. On day 28, the mice were killed, and the tumors were dissected, weighed and photographed. The tumor tissue was formalin fixed and paraffin embedded for subsequent immunohistochemical staining. In addition, the same number of cells was injected intraperitoneally with lentivirus to establish an intraperitoneal metastasis model. After 4 weeks, the mice were sacrificed and dissected. The number of metastatic nodules on the intra-abdominal tissue was observed. The metastatic tissues were stored at ‒80°C for protein analysis.

### Blood sampling

A standardized procedure was used to collect blood samples. After obtaining patient approval, we investigated 58 EOC patients and 28 healthy volunteers. Blood samples were drawn from patients at different treatment stages and from healthy volunteers and were subsequently centrifuged in a blood collection tube at 4°C. The serum was subsequently stored at ‒80°C until further use.

### ELISA

Serum BGN levels were measured via a sandwich ELISA kit (Invitrogen). A total of 100 μL of the sample to be tested was added to each sample, capped and incubated on a shaker for 2.5 h at room temperature. The solvent was discarded and washed 4 times with wash buffer, and 100 μL biotin was added to each well. The plates were placed on a shaker and incubated for 1 h at room temperature. The solvent was discarded and then washed. Streptavidin-HRP was added to each well. The plates were placed on a shaker and incubated for 45 min at room temperature. The solvent was discarded and then washed. After adding 100 μL TMB to each well, the matrix turned blue. The plates were placed on a shaker and incubated for 30 min at room temperature in the dark. A total of 50 μL of termination solution was added to each well, and the color of the solution changed from blue to yellow by gently tapping the side of the 96-well plate to mix. The corresponding optical density was measured at 450 nm.

### Statistical analysis

All the data are shown as the mean ± standard deviation (SD) from 3 independent experiments. Two-tailed Student’s unpaired *t* test was used for comparisons. Statistical analysis was performed via SPSS 21.0 and GraphPad Prism 5.0. The correlation between the expressions of PDK1 and BGN was evaluated via Spearman’s rank-order test. A value of *P*  < 0.05 indicated statistical significance.

## Results

### *PDK1* silencing inhibits EOC cell proliferation, migration and invasion

**Figure 1:**
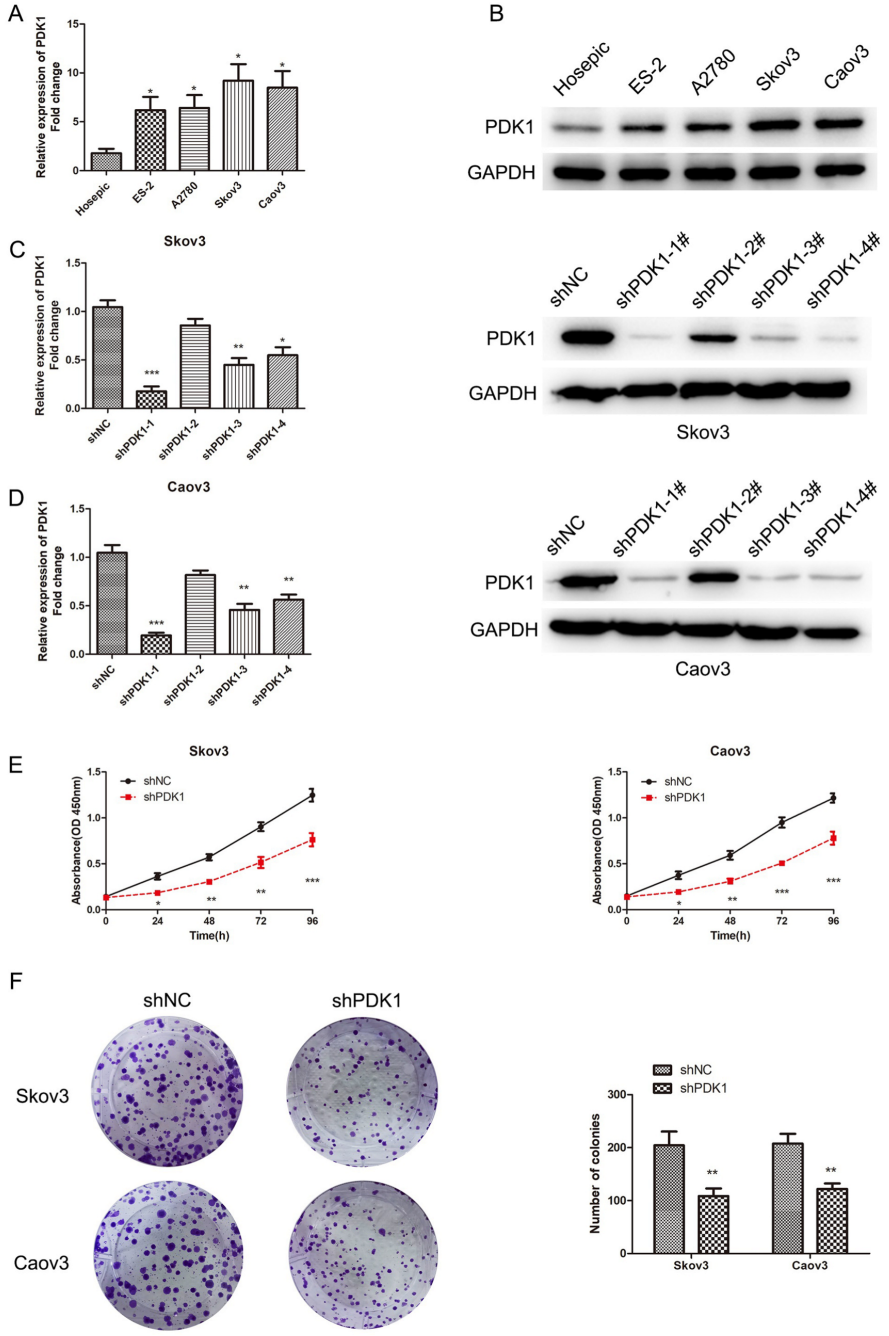
Figure 1 PDK1 knockdown represses EOC cell proliferation (A,B) PDK1 expression in Hosepic and EOC cell lines was determined. (C,D) PDK1 expression in EOC cells after transfection with shPDK1 lentivirus. (E) CCK-8 assay was used to detect cell viability after PDK1 was silenced in EOC cells. (F) Colony formation assay was used to detect cell growth after PDK1 was silenced in Skov3 and Caov3 cells. *P < 0.05, **P < 0.01, ***P < 0.001.

**Figure 2:**
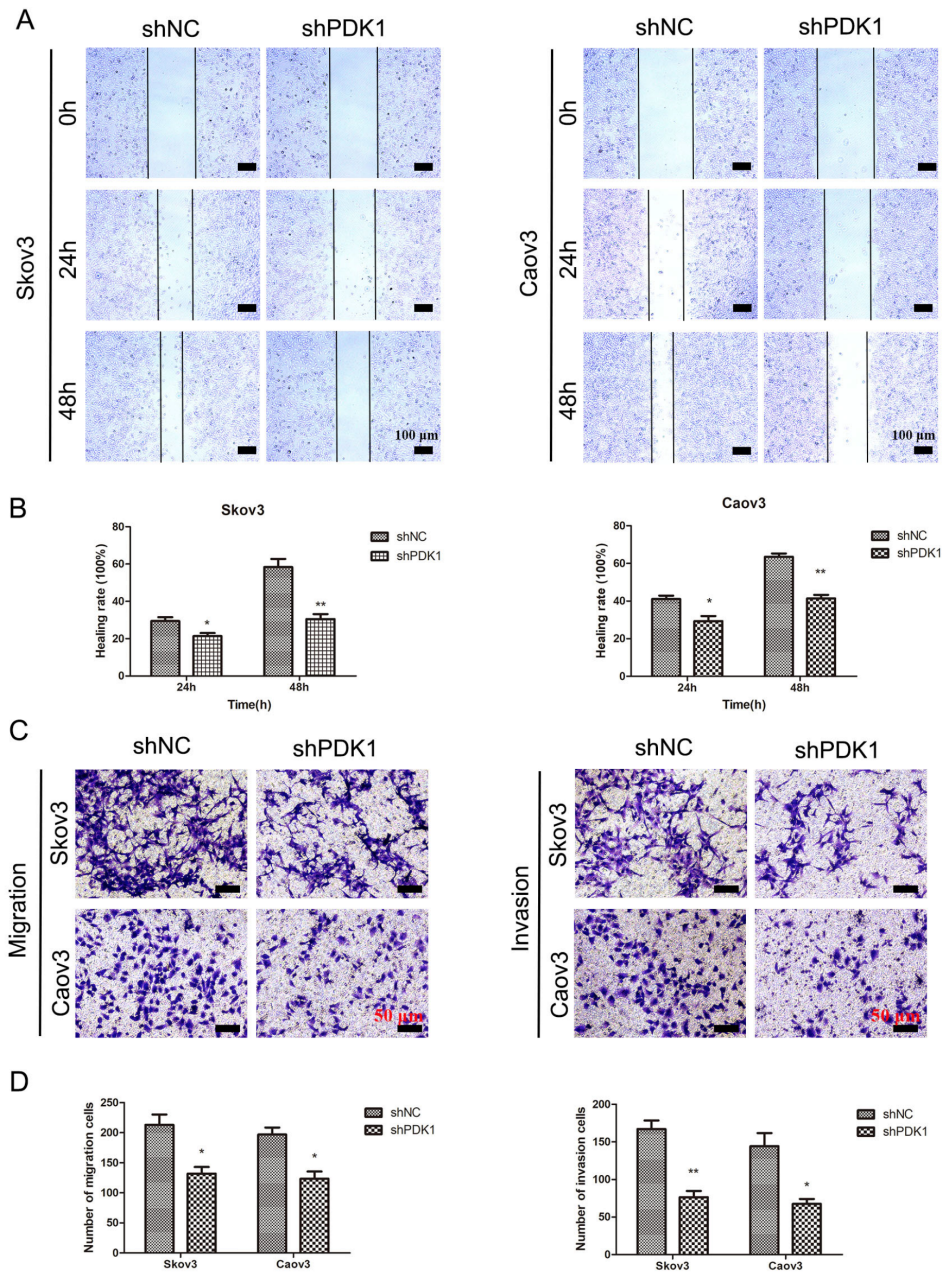
Figure 2 *PDK1* knockdown represses EOC cell migration and invasion (A,B) Wound healing assays were used to detect cell migration after PDK1 silencing in EOC cells (scale bar: 100 μm). (C,D) Transwell experiments were used to assess EOC cell migration and invasion after PDK1 was silenced (scale bar: 50 μm). *P < 0.05, **P < 0.01.

We assessed PDK1 expression in Hosepic, ES-2, A2780, Caov3 and Skov3 cells ( Figure 1A,B). Notably, PDK1 mRNA and protein expressions were elevated in Caov3 and Skov3 cells. Hence, these two cell lines were chosen to produce stable *PDK1*-silenced cells (Caov3-shPDK1 and Skov3-shPDK1). Following transfection, we used four distinct shRNA constructs to effectively decrease PDK1 expression and observed various degrees of knockdown efficiency. Among these, the shPDK1-1 construct significantly reduced PDK1 expression in both cell lines, which was subsequently chosen for detailed analysis ( Figure 1C,D). CCK-8 and colony formation assays revealed that cell proliferation in PDK1-depleted cells was significantly repressed ( Figure 1E,F). Furthermore, wound-healing experiments revealed a marked reduction in the migratory capacity of cells with downregulated PDK1 expression ( Figure 2A,B). Additionally, transwell assays demonstrated that PDK1 inhibition significantly inhibited EOC cell migration and invasion ( Figure 2C,D). Collectively, these findings indicated that the suppression of PDK1 expression attenuates the proliferative, migratory, and invasive potential of EOC cells *in vitro*, highlighting the pivotal role of PDK1 in the pathophysiology of EOC.

### *BGN* is a downstream target gene of *PDK1*

**Figure 3:**
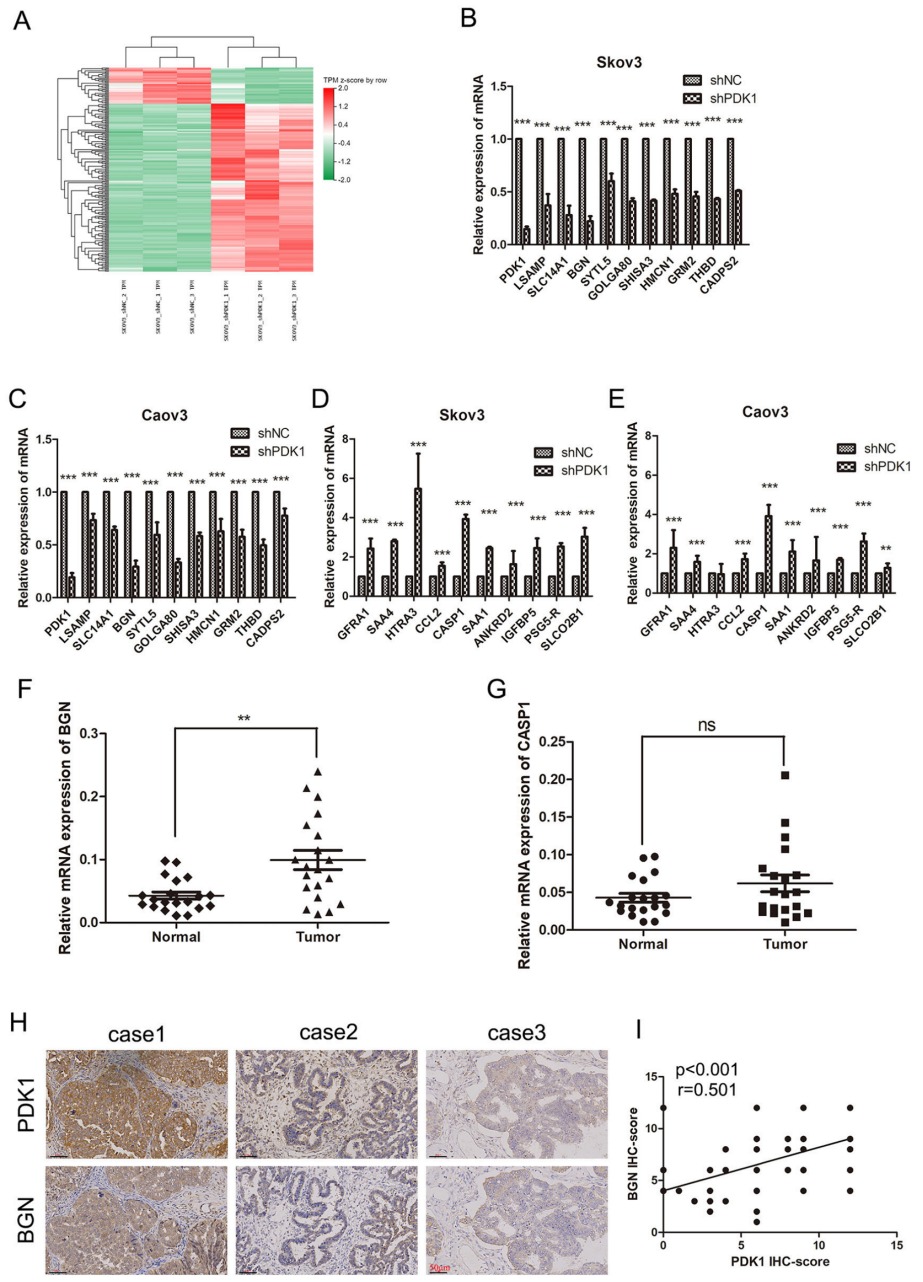
Figure 3 PDK1 expression is positively correlated with BGN in EOC tissues (A) Hierarchical clustering heatmap showing genes that are differentially expressed between control and shPDK1-transfected Skov3 cells. (B‒E) The effects of PDK1 silencing on the top ten DEGs were measured by qRT-PCR in Skov3 and Caov3 cells. (F) BGN expressions in 24 EOC tumor tissues and 24 related normal tissues were examined by qRT-PCR. (G) CASP1 expressions in 24 EOC tumor tissues and 24 related normal tissues were analyzed by qRT-PCR. (H) PDK1 and BGN expressions in EOC tissues were examined by immunohistochemistry (IHC) (scale bar: 50 μm). (I) Statistical analysis revealed a positive correlation between PDK1 and BGN in 60 EOC tissues. **P < 0.01, ***P < 0.001. ns, not significant.

To explore the downstream mechanism by which PDK1 accelerates EOC progression, RNA-Seq analysis was used to compare the gene expression profiles of Skov3 cells transfected with or without shPDK1. We identified 215 differentially expressed genes (DEGs), comprising 38 downregulated and 177 upregulated genes, between control and shPDK1-transfected Skov3 cells ( Figure 3A and Supplementary Figure S1A). According to the GO analysis, these DEGs were enriched in terms such as positive regulation of cell migration, gene expression, apoptotic process, extracellular matrix organization, epithelial-mesenchymal transition, protein phosphorylation, and negative regulation of cell proliferation, which also indicated that PDK1 dysregulation is responsible for the malignant phenotypes of ovarian cancer cells ( Supplementary Figure S1B). The results of the KEGG analysis revealed that these DEGs were enriched in cancer-related pathways, such as the TNF signaling pathway and the NF-κB signaling pathway ( Supplementary Figure S1C). Next, the top 10 upregulated and downregulated genes were selected for further analysis. qRT-PCR analysis revealed that *BGN* expression was most significantly reduced and that the expression of *CASP1* was most significantly elevated by silencing of *PDK1* in both Caov3 and Skov3 cells. Thus, BGN and CASP1 were screened for further research ( Figure 3B‒E). qRT-PCR analysis further revealed that *BGN* expression was significantly upregulated in EOC tissues, whereas *CASP1* expression was not significantly different between tumor and normal tissues. Thus, BGN was revealed to be regulated by PDK1 and potentially involved in EOC progression ( Figure 3F,G). To confirm the above results, PDK1 and BGN expression levels were further evaluated via immunohistochemistry in EOC tissues. Our results demonstrated that PDK1 and BGN expression levels were positively correlated in EOC tissues (r = 0.501, *P *​< 0.001) ( Figure 3H,I).

**Figure 4:**
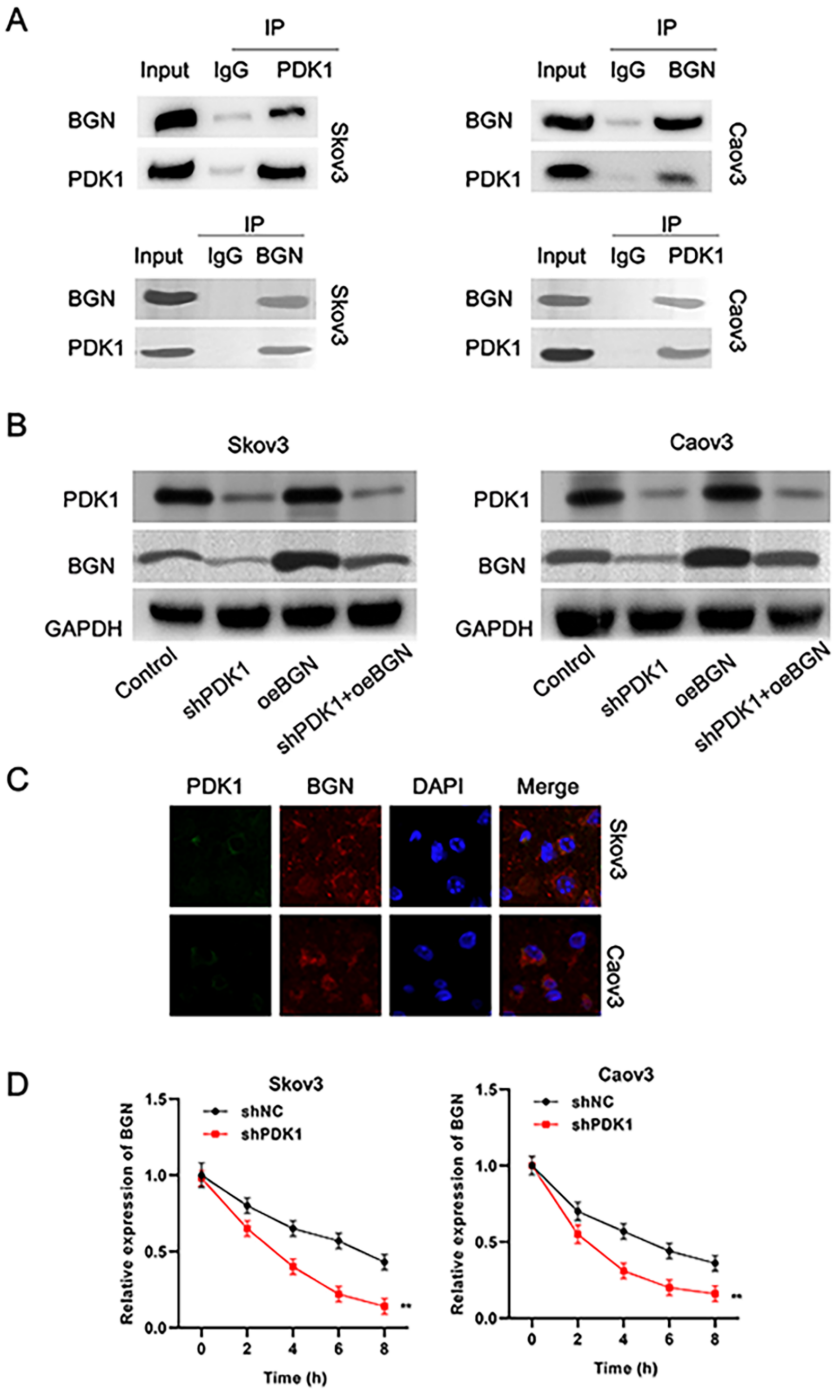
Figure 4 PDK1 binds to BGN and positively regulates BGN expression in EOC cells (A) Co-IP assays were conducted to evaluate the interaction between PDK1 and BGN in EOC cells. (B) Western blot analysis of the expressions of PDK1 and BGN in Skov3 and Caov3 cells after PDK1 knockdown or BGN overexpression. (C) FISH assay showing the colocalization of PDK1 and BGN in EOC cells. (D) qRT-PCR was used to detect the mRNA level of BGN in transfected EOC cells treated with actD. **P < 0.01.

Whether *BGN* is a downstream target of PDK1 in EOC cells was further explored. The Co-IP results verified that BGN interacted with PDK1 in EOC cells ( Figure 4A). Moreover, the protein expressions of PDK1 and BGN were significantly reduced after *PDK1* was silenced, whereas *BGN*overexpression caused the upregulation of BGN in only the oe-BGN or shPDK1 + oe-BGN groups compared with the control or shPDK1 groups, and PDK1 expression did not significantly change after *BGN* was overexpressed ( Figure 4B). Additionally, the results of the FISH assay revealed the colocalization of PDK1 and BGN in the cytoplasm of EOC cells ( Figure 4C). Next, we explored how PDK1 regulates *BGN* expression at the transcriptional level. We found that the decrease in the *BGN*mRNA level induced by actinomycin D was significantly elevated by *PDK1* knockdown in EOC cells ( Figure 4D). These findings indicated that *BGN* is a key downstream gene of PDK1 that affects EOC progression.

### BGN promotes EOC cell proliferation, migration and invasion

**Figure 5:**
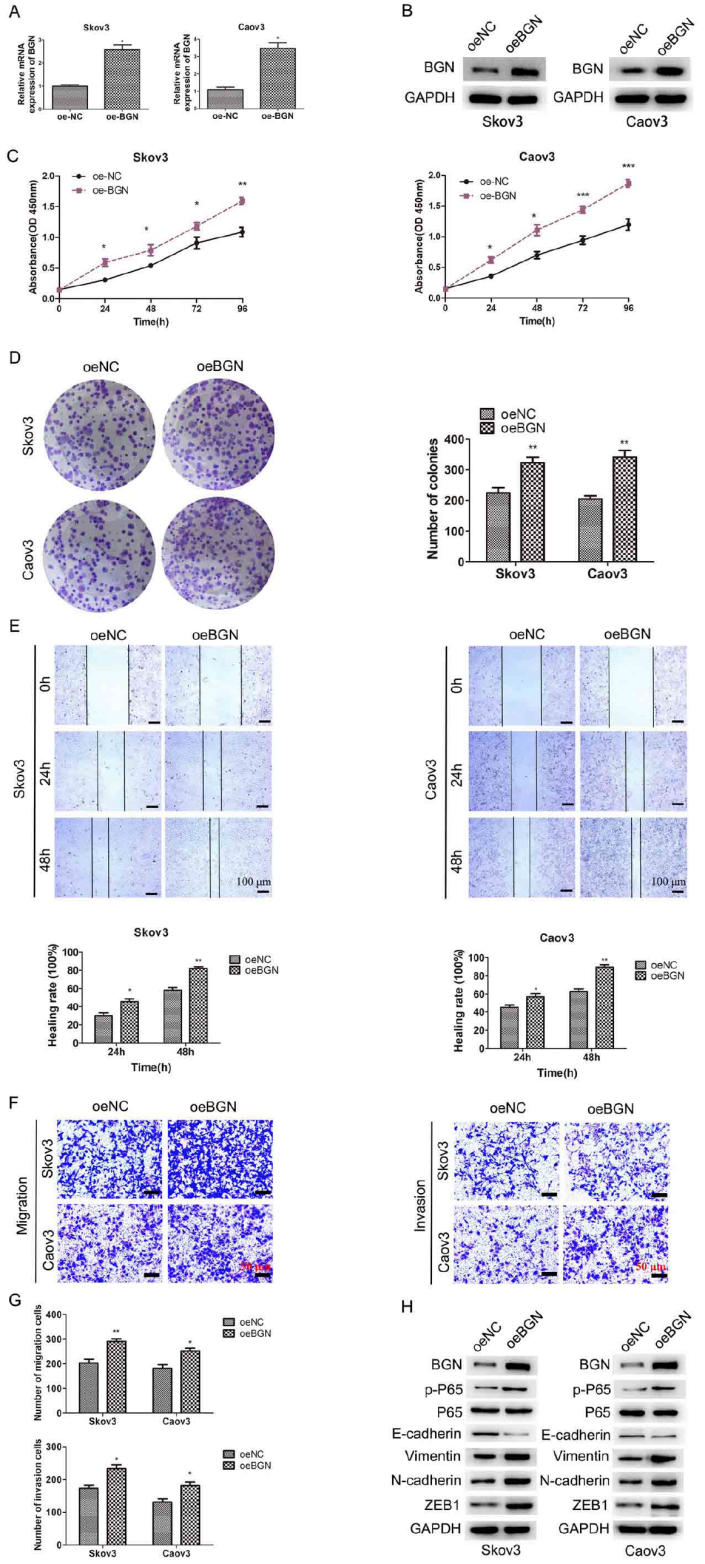
Figure 5 BGN promotes cell proliferation, migration, invasion and EMT in EOC (A,B) BGN expression in EOC cells after transfection with oeBGN lentivirus was examined. (C) CCK-8 assay was used to assess the viability of EOC cells overexpressing BGN. (D) Colony formation assays were used to detect the growth of EOC cells overexpressing BGN. (E) Wound-healing experiments were used to detect cell migration after BGN was overexpressed in EOC cells (scale bar: 100 μm). (F,G) Transwell experiments were used detected cell migration and invasion after BGN was overexpressed in EOC cells (scale bar: 50 μm). (H) Western blot analysis of the expressions of EMT-related and NF-κB-related proteins in EOC cells after BGN was overexpressed. *P < 0.05, **P < 0.01, ***P < 0.001.

To assess the biological effects of BGN on the growth and metastatic properties of EOC cells *in vitro*, two stable *BGN*-overexpressing cell lines (Caov3-oeBGN and Skov3-oeBGN) were established in the same way, with oeNC as a control. Our results revealed that transfection with oeBGN markedly increased *BGN* expression ( Figure 5A,B). CCK-8 assays revealed that EOC cell proliferation was increased in the *BGN*-overexpressing group ( Figure 5C). Moreover, the colony-forming ability of the oeBGN group increased significantly compared with that of the control group ( Figure 5D). Wound healing experiments revealed that the migration ability of Caov3-oeBGN or Skov3-oeBGN cells was clearly greater than that of Caov3-oeNC or Skov3-oeNC cells ( Figure 5E). Moreover, we observed that BNG overexpression significantly increased the migration and invasion potential of Caov3 and Skov3 cells ( Figure 5F,G). Our results suggested an oncogenic role of BGN in EOC progression and metastasis.

### BGN regulates EMT via the NF-κB pathway

EMT is closely associated with cancer cell migration and invasion and contributes to cancer metastasis [28]. Previous studies have demonstrated that BGN is correlated with the EMT process in multiple cancers [ 24, 29, 30] . BGN has also been reported to promote EMT via the NF-κB pathway in gastric cancer [29]. On the basis of the KEGG enrichment analysis, the DEGs in *PDK1*-silenced Skov3 cells were also enriched in the NF-κB signaling pathway ( Supplementary Figure S1C). Thus, we further investigated whether BGN regulates the activation of the NF-κB pathway and EMT in EOC. Western blot analysis was used to determine the relative levels of EMT markers, including E-cadherin, N-cadherin, vimentin, and ZEB1. The results revealed that increased BGN expression significantly reduced E-cadherin level, and elevated N-cadherin, vimentin and ZEB1 expression levels in EOC cells ( Figure 5H). These results indicated that BGN accelerates the EMT process and promotes EOC invasion and migration.

Furthermore, we assessed the total and phospho-p65 levels in EOC cells following *BGN* overexpression. Our results indicated that the overexpression of BGN did not affect total p65 expression. Interestingly, upregulated BGN significantly increased phospho-p65 expression. The above data demonstrated that BGN regulates EMT and activates the NF-κB signaling pathway in EOC cells, suggesting that BGN is a key factor in EOC progression.

### PDK1 upregulates BGN to activate NF-κB signaling and promotes EOC cell proliferation, migration and invasion

**Figure 6:**
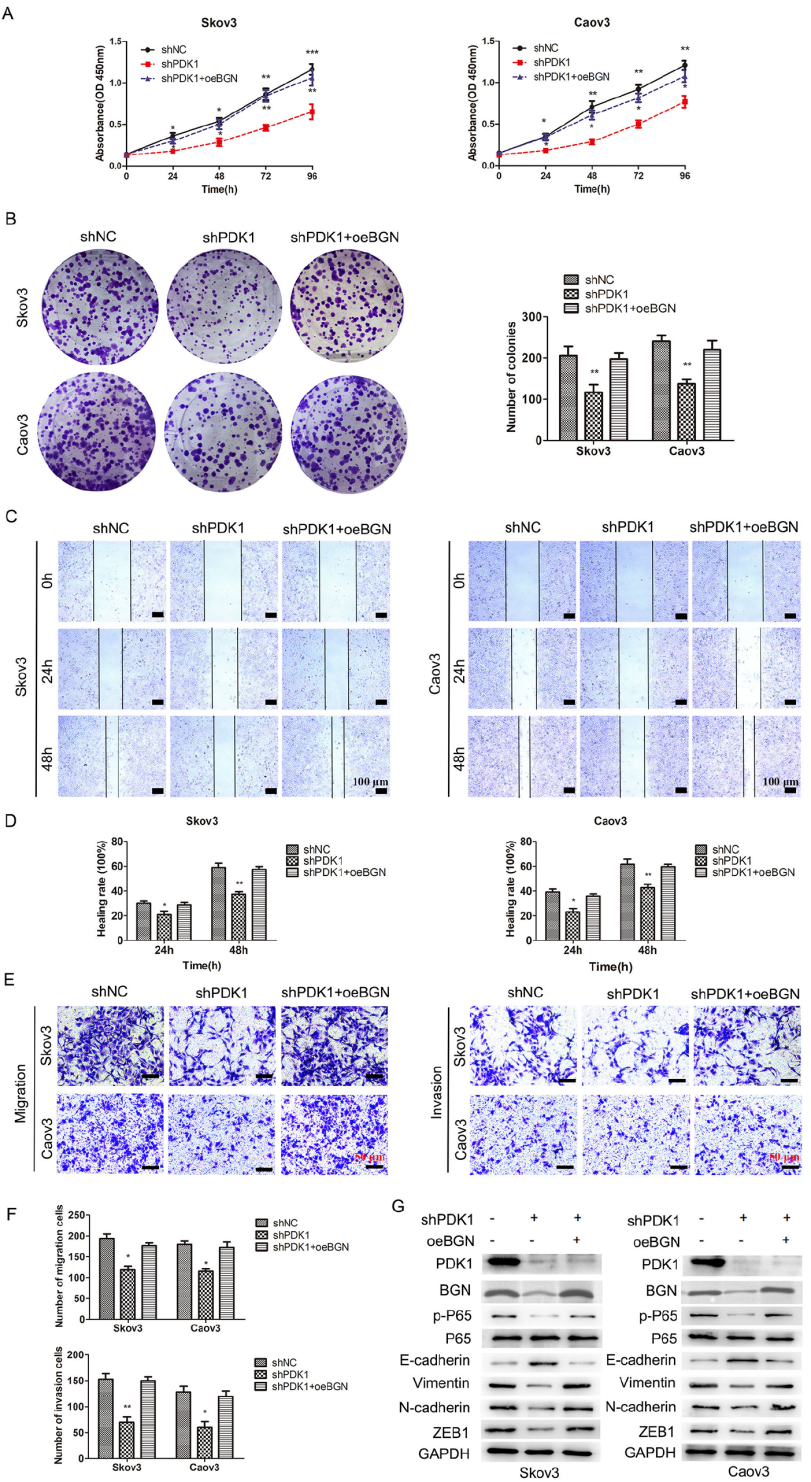
Figure 6 PDK1 mediates BGN-induced proliferation, migration, invasion and EMT (A) Effects of PDK1 downregulation and/or BGN overexpression on the proliferation of EOC cells were detected via CCK-8 cell proliferation assay. (B) Colony formation assay was used to detect the effects of PDK1 downregulation and/or BGN overexpression on Skov3 and Caov3 cell proliferation. (C,E) Wound-healing assays were used to detect Skov3 and Caov3 migration after the indicated transfections (scale bar: 100 μm). (D,F) Skov3 and Caov3 migration and invasion were detected by Transwell assays after the indicated transfections (scale bar: 50 μm). (G) Western blot analysis of EMT- and NF-κB-related proteins in EOC cells after the indicated transfections. *P < 0.05, **P < 0.01, ***P < 0.001.

To validate whether PDK1 regulates the malignant behaviors of EOC cells by targeting BGN, shPDK1 and oeBGN vectors were cotransfected into EOC cells. Co-transfection resulted in partial restoration of cell proliferation ( Figure 6A,B), migration capacity ( Figure 6C,D), and invasion capacity ( Figure 6E,F), which were reduced by *PDK1* knockdown alone. Further analysis of the NF-κB signaling pathway revealed a partial increase in BGN, N-cadherin, vimentin, ZEB1, and phospho-p65 expressions and a decrease in E-cadherin expression without altering total p65 level in the co-transfected group ( Figure 6G). Our findings suggested that *PDK1* silencing inhibits EOC cell proliferation, migration and invasion by reducing BGN expression, underscoring the therapeutic potential of targeting the PDK1-BGN-NF-κB axis.

### PDK1 facilitates tumor growth and metastasis *in vivo* by increasing BGN expression

**Figure 7:**
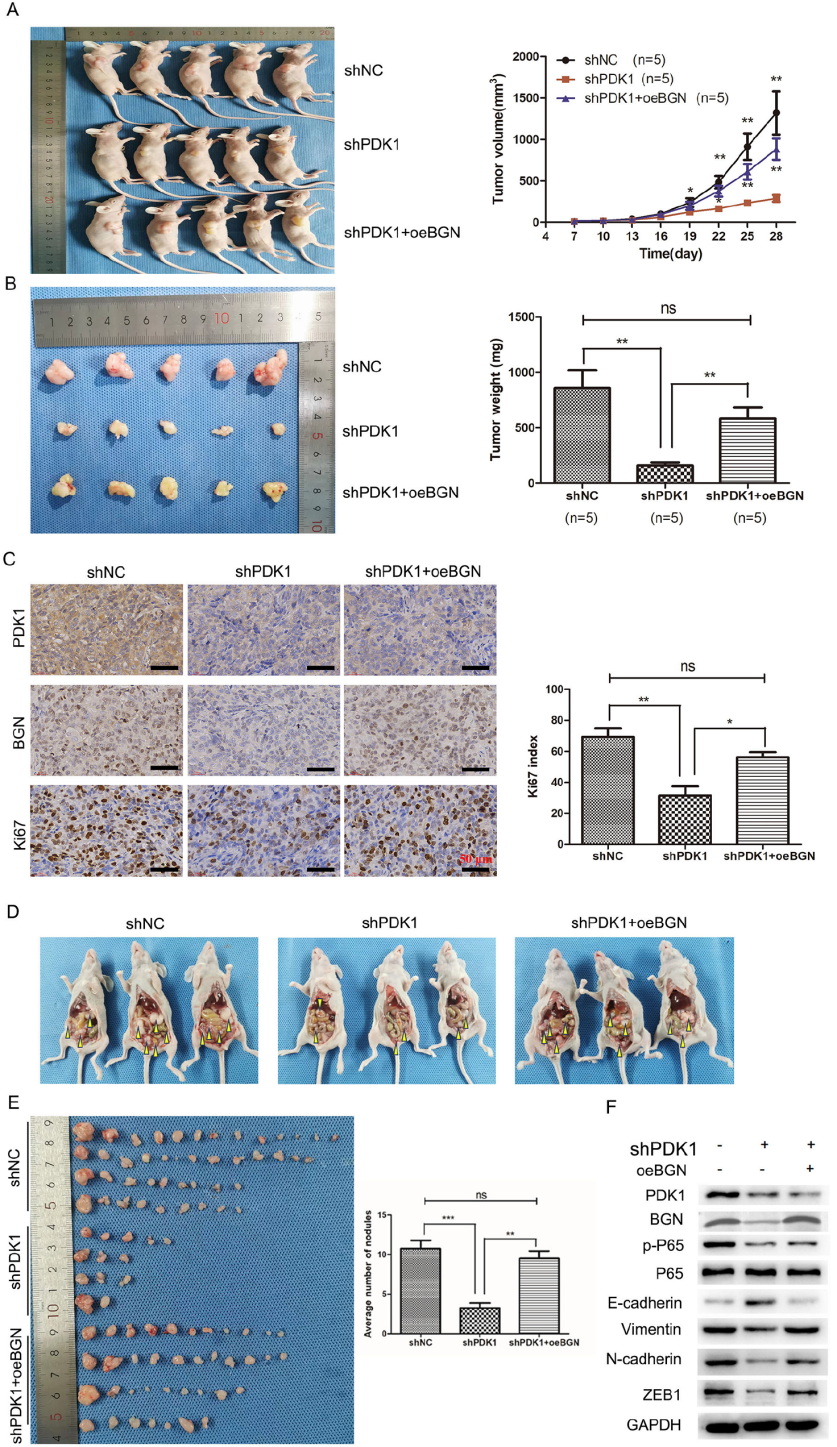
Figure 7 PDK1 mediates BGN-induced xenograft tumor growth and metastasis (A) Tumor growth curves of the shNC, shPDK1 and shPDK1 +oeBGN groups. (B) The average tumor weight in each group was measured. (C) Representative images of IHC staining with the indicated antibodies in tumors isolated from mice (scale bar: 50 μm). (D) Intraperitoneal metastasis model showing the metastatic nodules derived from the coinfected group, shPDK1 group and shNC group. (E) Intraperitoneal metastasis tumor weights and amounts were measured in each group. (F) The expressions of PDK1, BGN, NF-κB and EMT-related markers in tissues derived from xenograft tumors, as determined by western blot analysis. *P < 0.05, **P < 0.01. ns, not significant.

Since the increased level of PDK1 in EOC patients is related to metastasis [13], we hypothesized that PDK1 may increase the invasive and metastatic ability of EOC cells. Using a nude mouse xenograft model, we found that *PDK1* knockdown significantly inhibited ovarian tumor growth ( Figure 7A,B), and Ki-67 immunohistochemistry revealed a decreased proliferation index in tumors from *PDK1*-silenced mice compared with controls ( Figure 7C). Conversely, tumors in mice in the shPDK1 + oeBGN group were somewhat greater in size and weight than tumors in mice infected solely with shPDK1. Immunofluorescence data revealed that BGN and Ki-67 expressions were greater in nodules from the coinfected group than in nodules from the shPDK1 group. We further assessed the ability of the PDK1-BGN axis to promote metastasis via intraperitoneal injection in an intraperitoneal metastatic model. Our findings revealed that the total number of intraperitoneal metastatic nodules in the co-infected mice was much greater than that in the shPDK1 nude mice ( Figure 7D,E). Furthermore, western blot analysis revealed that E-cadherin protein level was markedly increased, whereas p-p65, N-cadherin, vimentin and ZEB1 levels were decreased in xenograft tumors from shPDK1 mice ( Figure 7F). Moreover, the above protein levels were partially recovered in xenograft tumors from co-infected mice. These results suggest that PDK1 promotes EOC tumorigenesis and metastasis via BGN regulation in *vivo*.

### Serum BGN is a diagnostic biomarker in EOC

**Figure 8:**
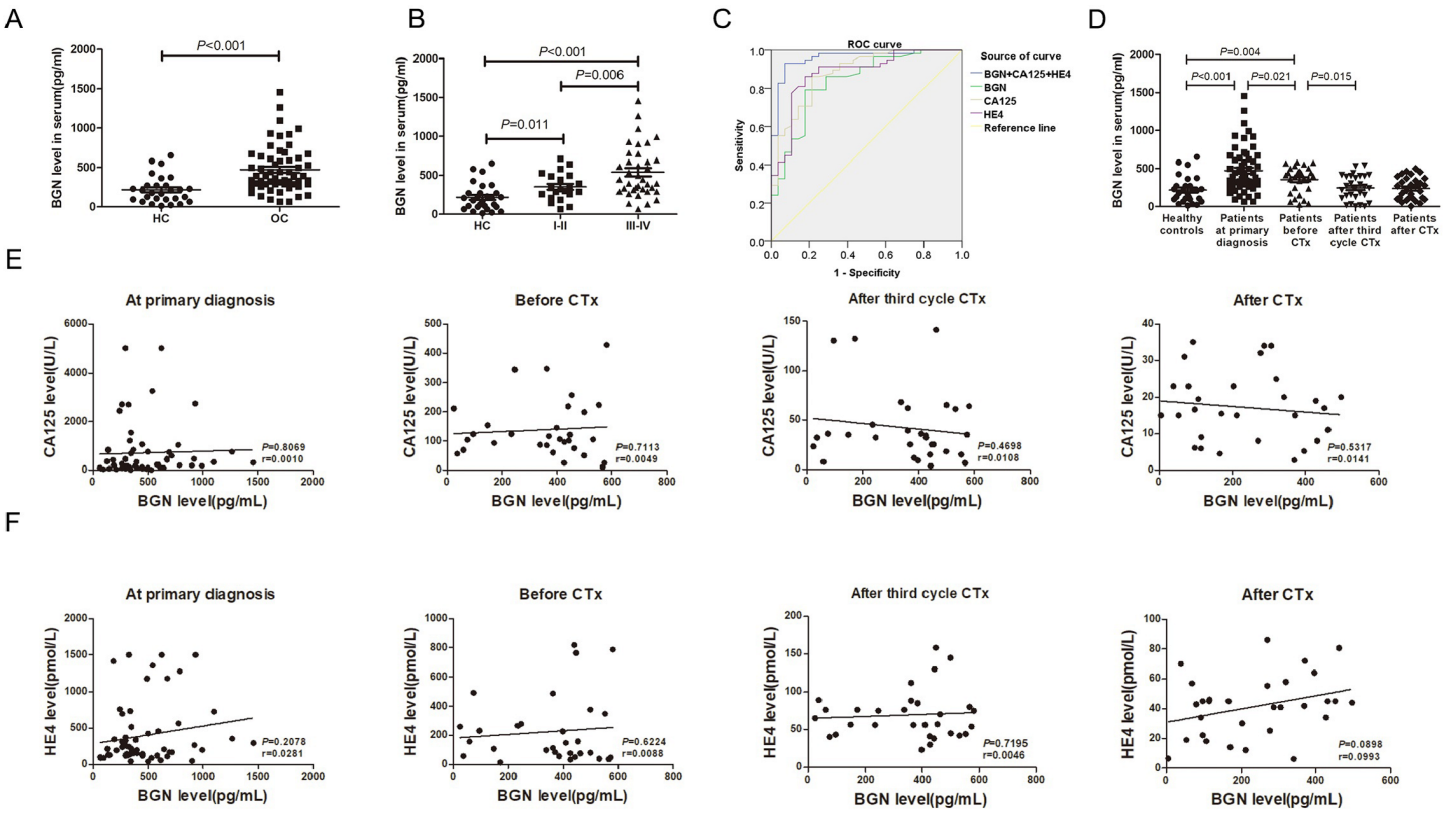
Figure 8 The serum BGN level is a potential biomarker for the diagnosis of EOC (A) BGN expression in the serum of EOC patients was significantly different from that in healthy controls. (B) BGN expression in the serum of low-FIGO stage patients (I + II) was different from that in the healthy controls. BGN expression in the serum was different between high-FIGO stage patients (III + IV) and low-FIGO stage patients (I + II). (C) ROC curve of the sensitivity versus specificity of BGN, CA125, and HE4, as well as the combination of the three markers. (D) The serum BGN concentration of EOC patients was detected at primary diagnosis (n = 58) and in three longitudinal serum samples from each patient before platinum-based chemotherapy (n = 30), after the third cycle of chemotherapy (n = 30), and after the completion of chemotherapy (n = 30). (E,F) Correlation of BGN and CA125/HE4 levels in peripheral blood collected at primary diagnosis, before platinum-based chemotherapy (CTx), after the third cycle of chemotherapy (CTx), and after the completion of chemotherapy (CTx).

Some reports have revealed that BGN expression in serum is linked to the malignancy of cancer [ 31, 32] . We detected the expression of BGN in the serum of healthy individuals and EOC patients. In contrast to that in the healthy controls, the concentration of BGN in the EOC group was greater ( Figure 8A). Additionally, the BGN level in the FIGO III--IV subgroup was significantly different from that in the FIGO I--II subgroup ( Figure 8B). Analysis of serum BGN levels in relation to clinicopathological features in 58 patients revealed a correlation with advanced FIGO stage ( Table 1). The diagnostic performance of BGN in EOC was further explored via ROC curve analysis. CA125 and HE4 are commonly used diagnostic biomarkers in EOC. As shown in Table 2, the ROC curve revealed that the BGN concentration in EOC was 0.837, the cut-off value was 280.84 pg/mL, the sensitivity was 0.793, and the specificity was 0.821. The ROC curve for CA125 in EOC diagnosis revealed that the AUC = 0.882, the cut-off value was 41.5 U/L, the sensitivity was 0.862, and the specificity was 0.786. The ROC curve for EOC diagnosis by HE4 concentration indicated that the AUC = 0.881; the cut-off value was 105.5 pmol/L; the sensitivity was 0.862; and the specificity was 0.821. The results also revealed that the AUC was 0.959, the sensitivity was 0.931, and the specificity was 0.929 with the combined detection of BGN, CA125, and HE4 ( Figure 8C). These results suggest that BGN may be a potential diagnostic biomarker and that the combined detection of BGN, CA125, and HE4 has good performance in the diagnosis of EOC.

**Table:** **Table 1** Clinicopathological variates in 58 patients according to BGN level in preoperative serum

Clinicopathologic features	Low blood BGN ( *n* = 29)	High blood BGN ( *n* = 29)	χ ^2^	*P* value
Age (year)				
< 56	17	15	0.336	0.386
≥ 56	12	14		
Size (cm)				
< 8	13	15	0.279	0.792
≥ 8	16	14		
FIGO stage				
I‒II	15	6	6.046	0.028*
III‒IV	14	23		
Differentiation				
G1‒G2	17	13	1.105	0.2932
G3	12	16		
Lymph node metastasis			
No	17	11	2.486	0.189
Yes	12	18		

* *P* < 0.05.

**Table:** **Table 2** Receiver operating characteristic (ROC) curve of serum BGN levels for the diagnostic ability of ovarian cancer

	Cut-off	YOUDEN	Sensitivity	Specificity	AUC	95%CI
						lower	upper
BGN (pg/mL)	280.84	0.614	0.793	0.821	0.837	0.745	0.929
CA125 (U/L)	41.50	0.648	0.862	0.786	0.882	0.806	0.958
HE4 (pM)	105.50	0.683	0.862	0.821	0.881	0.802	0.959
Combined	–	0.860	0.931	0.929	0.959	0.916	0.999

Furthermore, we investigated the value of BGN in assessing therapeutic response. The serum of 30 patients after treatment was collected, and the serum BGN levels in EOC patients after therapy were measured. Interestingly, compared with those before treatment, the serum BGN levels decreased after therapy, especially after surgery and adjuvant chemotherapy ( Figure 8D). In addition, the circulating BGN level in the serum of EOC patients was mostly independent of the CA125/HE4 serum level ( Figure 8E,F). Clinical results indicate that detecting serum BGN, especially in conjunction with CA125 and HE4, is a valuable biomarker for diagnosing and monitoring EOC treatment response.

## Discussion

EOC has been identified as the primary cause of death from gynecological cancer [33]. Effective diagnosis and treatment measures are needed to reduce the risks and improve the clinical outcomes of this aggressive malignancy [34]. PDK1, a pyruvate dehydrogenase kinase family member related to oncogenic pathways, is overexpressed in diverse cancers and is valuable in targeted cancer therapy [35]. In this study, we found that PDK1 had an oncogenic effect on EOC cells and Skov3 xenografts by modulating BGN, which might increase the understanding of the molecular basis of EOC.

PDK1 is critically involved in regulating cancer cell growth, survival, metastasis and metabolism [36]. PDK1 expression is associated with oncogenic activity in cancer progression. Previous studies have reported that PDK1 is highly expressed in ovarian cancer and acts as an oncogene in ovarian cancer via diverse mechanisms. For example, PDK1 facilitates ovarian cancer cell migration, invasion, and angiogenesis by regulating α5β1 integrins and activating JNK/IL-8 signaling [37]. Moreover, PDK1 has also been revealed as an outstanding predictor of ovarian cancer prognosis, and patients with high PDK1 expression have a reduced overall survival rate [13]. Our previous findings suggested that PDK1 may be a prognostic factor for EOC patients [13]. In this study, PDK1 depletion significantly repressed EOC cell proliferation, migration and invasion. Furthermore, *PDK1* knockdown markedly reduced the tumor burden and peritoneal metastasis of Skov3 cells in mice. These results strongly corroborate the substantial oncogenic capacity of PDK1, aligning with previous reports and confirming its therapeutic target potential.

The downstream regulatory mechanism of PDK1 was further explored. Our study revealed that BGN is a downstream gene of PDK1 in EOC. BGN is correlated with the extracellular matrix under physiological conditions and is also expressed on the cell surface [ 38, 39] . BGN is highly expressed in multiple tumors, including pancreatic cancer, esophageal cancer, gastric cancer, colon cancer, endometrial cancer and prostate cancer, and transcriptional analysis also revealed that BGN is a promising diagnostic and therapeutic target in different cancers [ 30, 40] . Additionally, as a powerful signaling molecule, BGN affects tumor development via a variety of signaling pathways [ 41‒ 45] . Manupati *et al*. [19] reported that BGN accelerates breast cancer cell proliferation and invasion via the NF κB pathway. Moreover, a previous study showed that BGN can induce EMT in a variety of malignant tumors [46]. When EMT occurs, the ability of cancer cells to metastasize and migrate to neighboring cells increases [ 47, 48] . EMT thereby has critical potential in cancer metastasis [ 49, 50] .

In this study, we found that PDK1 interacts with BGN and positively regulates BGN expression by modulating BGN protein stability in ovarian cancer cells. Similarly, HSPA8 reduces CLPP protein stability in ovarian cancer cells and affects ovarian cancer cell drug resistance and survival via CLPP [51]. *BGN* overexpression enhanced EOC cell proliferation, migration, invasion, and EMT *in vitro*. BGN upregulation increased the level of p-p65/p65 in EOC cells. Dysregulation of the NF κB signaling pathway is critically associated with cancer progression [52]. NF κB p65 is a key member of the NF κB family and is reported to initiate the transcription of factors involved in EMT induction [53]. Knockdown of NF κB p65 suppresses the migration and invasion of ovarian cancer cells [54]. The inhibition of EMT has the potential for preventing drug resistance and enhancing the anticancer effects of therapy [55]. Importantly, our findings revealed that BGN overexpression reversed the inhibitory effects of *PDK1* knockdown on p-p65/p65 levels in EOC cells. BGN upregulation also partially rescued the impacts on proliferation, wound-healing ability, migration, invasion and EMT caused by *PDK1* knockdown. Additionally, we found that *PDK1* silencing inhibited the subcutaneous tumor growth and peritoneal metastasis of EOC in mice, whereas increased BGN partially reversed these results. Hence, our work may offer novel insights into the mechanisms involving the PDK1-BGN-NF κB axis. Additionally, *BGN*, a downstream gene of PDK1, can be detected in serum and is regarded as a useful indicator in various diseases [ 56, 57] .

In our study, BGN expression in the serum of EOC patients was greater than that in the serum of healthy controls and was significantly associated with FIGO stage. Compared with each biomarker alone, the combined biomarkers of BGN, CA125, and HE4 had better performance in diagnosing EOC. Moreover, the serum BGN level was also significantly lower after the third cycle of chemotherapy than before platinum-based chemotherapy, while no significant correlation was found between the serum BGN level and CA125/HE4 level after diagnosis and treatment, suggesting that BGN is an independent biomarker for therapeutic response in EOC patients. This result also strongly suggests that the PDK1-BGN axis may be used as a therapeutic target for EOC.

Nevertheless, this study has several limitations. The NF κB pathway was demonstrated to be regulated by the PDK1-BGN axis, but the effects of pathway activation on PDK1- or BGN-silenced EOC cells need to be validated in the future. Additionally, mouse models were established using human Skov3 cells, which cannot reflect the impact of the tumor microenvironment on PDK1 or BGN during EOC progression. Future work is expected to build 3D tumor models to investigate the interaction between EOC cells and the microenvironment and the effects of the PDK1-BGN-NF κB axis on tumor progression.

In summary, our findings revealed that *PDK1* silencing inhibits the EMT process, migration, and invasion of EOC cells, as well as tumor growth and metastasis, in mice by regulating BGN. Serum BGN is significantly elevated in EOC and has robust diagnostic value. The PDK1-BGN axis is suggested as a therapeutic target for preventing and intervening EOC metastasis.

## Supporting information

24361Supplementary_Figure_S1

## Supplementary Data

Supplementary data is available at *Acta Biochimica et Biophysica Sinica* online.
