# Supplementary material for: PDK1 promotes epithelial ovarian cancer progression by upregulating BGN: PDK1/BGN axis facilitates EOC progression
Source: Acta Biochim Biophys Sin (Shanghai). 2024 Nov 21;57(5):712–26. doi: 10.3724/abbs.2024186 (PMC12130725; doi:10.3724/abbs.2024186)
Supplement: 24361Supplementary_Figure_S1 [file 24361Supplementary_Figure_S1.docx]

**
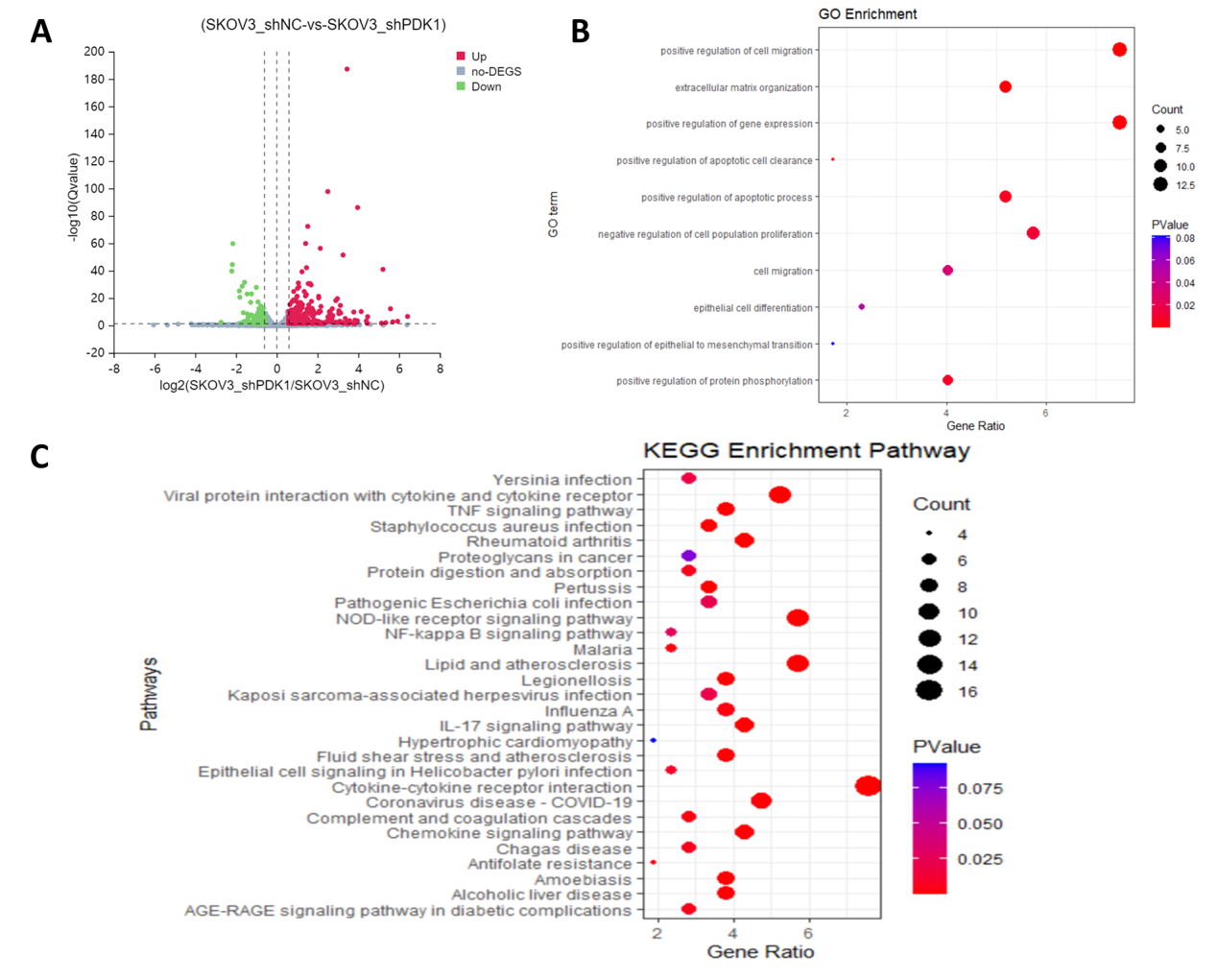
**

**Supplementary Figure S1. Enrichment analysis of the DEGs in shPDK1-transfected Skov3 cells** (A) Volcano plot showing the upregulated (red color) and downregulated genes (green color) in shPDK1-transfected Skov3 cells. (B) GO and (C) KEGG enrichment analyses of the DEGs in shPDK1-transfected Skov3 cells.
